# Smart Biopolymer Scaffolds Based on Hyaluronic Acid and Carbonyl Iron Microparticles: 3D Printing, Magneto-Responsive, and Cytotoxicity Study

**DOI:** 10.1021/acsabm.4c00567

**Published:** 2024-10-17

**Authors:** Danila Gorgol, Miroslav Mrlík, Filip Mikulka, Zdenka Víchová, Leona Mahelová, Markéta Ilčíková, Antonín Minařík

**Affiliations:** † Centre of Polymer Systems, Tomas Bata University in Zlin, Trida T. Bati 5678, 760 01 Zlin, Czech Republic; ‡ Department of Physics and Materials Engineering, Faculty of Technology, Tomas Bata University in Zlin, Vavřečkova 275, 70 01 Zlin, Czech Republic; § Polymer Institute, Slovak Academy of Sciences, Dubravska cesta 9, 845 45 Bratislava, Slovakia

**Keywords:** scaffold, hyaluronic acid, magnetic particles, 3D printing, magneto-responsive

## Abstract

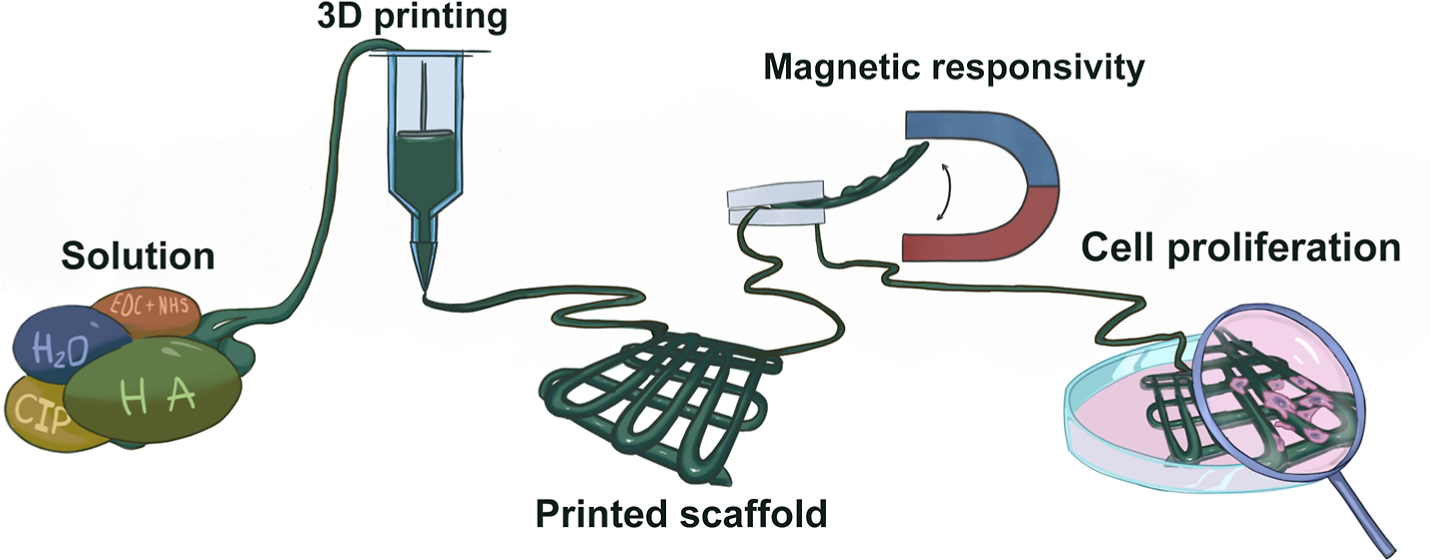

This study deals with utilization of the hyaluronic acid (HA) and carbonyl iron (CI) microparticles to fabricate the magneto-responsive hydrogel scaffolds that can provide triggered functionality upon application of an external magnetic field. The various combinations of the HA and CI were investigated from the rheological and viscoelastic point of view to clearly show promising behavior in connection to 3D printing. Furthermore, the swelling capabilities with water diffusion kinetics were also elucidated. Magneto-responsive performance of bulk hydrogels and their noncytotoxic nature were investigated,, and all hydrogels showed cell viability in the range 75–85%. The 3D printing of such developed systems was successful, and fundamental characterization of the scaffolds morphology (SEM and CT) has been presented. The magnetic activity of the final scaffolds was confirmed at a very low magnetic field strength of 140 kA/m, and such a scaffold also provides very good biocompatibility with NIH/3T3 fibroblasts.

## Introduction

The scaffold in the most cases is a typical three-dimensional construct with properly interconnected pores.^1^ In general, this structure serves as a platform for tissue growth.^2^ It can be interpreted as a hydrogel in the case when it has a low cross-linking degree, high porosity, and moderate water absorption.^3,4^ In addition, it can be used as a drug delivery device or a growth factor.^5,6^ Every cell type thrives in the specific conditions and grows on the most suitable surface; because of this, the structure of the scaffold has to mimic the original cell environment.^7,8^ This 3D object has an enormous potential to be modified according to the goals. Scaffolds with suitable porosity combined with bioactive factors lead to the success of cell attachment and further proliferation.^1,9,10^

There are a lot of ways to fabricate scaffolds. Among approximately 40 different 3D-printing techniques, fused deposition modeling, stereolithography, inkjet printing, selective laser sintering, melt electro-writing,^11,12^ and colorjet printing appear to be the most popular.^13^

Hydrogels have dualistic behavior when saturated with water; hydrogels behave like both solids and fluids. Due to a thin cross-linked polymer network, hydrogels show the properties of elastic solids with deformability and softness.^14,15^ However, the dominant water amount inside the swelled up system makes the whole hydrogel have liquid-like attributes, such as permeability to a wide range of chemical and biological agents. For tissue engineering and regenerative medicine applications, it can be used as a cell matrix with biocompatible and biodegradable support and native tissue-like attributes.^16,17^

Hydrogels with magnetic particles were already studied in the work of Bin et al. The poly(acrylamide) hydrogel with incorporated carbonyl iron (CI) powder in order to create soft robotics was utilized.^18^ Kondo and Fukuda, fabricated synthetic hydrogel with magnetite particles for an application in enzyme immobilization.^19^ In general, a magnetic hydrogel is a great candidate for cell cultivation and cell differentiation in a magnetic field. The magnetic sensitivity provides a possibility to deform and change the geometry of the 3D printed scaffold according to magnetic field. This allows inserted stem cells to successfully grow and transform into muscle tissue depending on motion in the presence of a magnetic field.^20−25^ The CI particles were found to be noncytotoxic even before various coatings^26,27^ and also do not provide any significant decrease of cell viability over 48 h when they are used in composite scaffolds.^12^ Also, the high magnetic saturation of such particles is very promising; mixing them with the scaffold enables to provide a magnetically active biosystem.^28−30^

A spectrum of various polymers was used for scaffold fabrication. It contains a range of natural [collagen, silk, alginate, chitin, starch, and hyaluronic acid (HA)],^31−33^ and synthetic polymers [poly(lactic acid), poly(caprolactone), and poly(urethane)]^34^ that are presented in the system independently or in their combination to improve final scaffold applicability. In this work, as a base for hydrogel, HA was chosen.

HA or sodium hyaluronate (SH) in the stabilized form is a polysaccharide, naturally present in almost all life forms.^35^ The unique viscoelastic properties of HA in combination with its high biocompatibility make it a potential candidate for use in clinical applications, including the supplementation of joint fluid, eye surgery, and regeneration of surgical wounds.^35,36^ Regardless of all the mentioned excellent bioactive properties and notable biocompatibility, pure HA without any modification is not in many cases suitable for 3D printing, since it exists as a mostly viscous solution that is not stable after the printing process. Fortunately, the stability of HA can be changed and upgraded through chemical and physical modifications.^37,38^ In many research papers, a stabilization process was cross-linked with *N*,*N*-(3-(dimethylamino)propyl)-*N*′-ethyl-carbodiimide hydrochloride (EDC) and *N*-hydroxysuccinimide (NHS). This method was successfully applied on HA and collagen.^39−41^ However, in this work, cross-linking between HA molecules was achieved without significant pH regulation. Moreover, there are some recent papers utilizing the 3D printing of HA and magnetic particles; however, in both cases, based on the magnetic nanoparticles, those magneto-responsive characteristics are limited, due to their low magnetization saturation.^42,43^ In the majority of the cases, publications dealing with HA and magnetic particles even refer to magnetic microparticle domains; however, they are, from the magnetic point of view, iron oxide nanoparticles.^44−46^ The utilization of the magnetic microparticles, as presented in this study, and HA was reported recently;^47^ however, in this case, the HA was modified and there is no 3D printing.

Based on the all mentioned limitations, in this study, we have developed and fabricated a smart hydrogel scaffold based on cross-linked HA containing CI with very good shape fidelity and stability in the aqueous medium as well as in cultivation medium. Moreover, such cross-linked HA-CI systems are suitable for 3D printing due to the suitable shear-thinning capabilities and also provide excellent magnetic activity in moderate magnetic field strengths (140 kA/m). Moreover, all investigated hydrogels containing 10, 20, and 30 wt % magnetic particles show cell viability classified as nontoxic and scaffold with 30 wt % of CI shows good cell proliferation in direct contact.

## Materials and Methods

The HA was in the form of SH (HA, Contipro Biotech, Czech Republic). The HAs of three different molecular weights were used. The molecular parameters were determined by the AF4-MALS chromatography,^48^ and they were adopted from the literature. The average number and weight molar mass and dispersity are listed in Table1. The magnetic carbonyl iron particles (CI; CN grade, iron content minimum of 99.5%, d50 = 6.5–8.0 μm) (BASF, Germany) with magnetization saturation 203.2 emu/g were used without surface modification. These particles exhibit almost negligible coercivity as well as remnant magnetization and therefore are very promising candidates as magneto-active species.^49^ The EDC and NHS were purchased from Sigma-Aldrich and used as received. Distilled water used in this study was prepared using a Simplicity UV water purification system (Milli-Q, Merck).

**Table 1:** Molecular Parameter of HA (Number and Weight Molecular Weight Average *M*_n_ and *M*_w_) and Dispersity *D̵*

sample	*M*_n_ (kDa)	*M*_w_ (kDA)	*D̵*
HA1	161.8	186.2	1.15
HA2	377.0	473.3	1.26
HA3	978.6	998.3	1.02

### Sample Preparation

The hydrogel samples were prepared in several steps. The general procedure is depicted in Figure S1 and is described as follows: the HA (0.3 g, 8.11 × 10^–4^ mmol) was dissolved in demineralized water (2.7 mL) to obtain 7 wt % HA solution. The HA was dissolved using magnetic stirring for 24 h. Then the CI particles (0.93 g) were added to prepare the sample with 30 wt % of particles. The mixture was stirred for another 4 h at room temperature. Finally, the components of cross-linking system EDC (0.045 g, 0.29 mmol) and NHS (0.045 g, 0.39 mmol) were added gradually. Then, the cross-linking reaction was completed within 50 min at ambient temperature. In this work, various conditions were tested in terms of concentration of HA solution (1, 4, and 7 wt %) and concentration of CI particles (10, 20, and 30 wt %). During all the preparations, the pH was not additionally corrected and measured pH was, for all formulations, in the range of 7.3 to 7.9.

### Scaffold Fabrication

The process was performed by a 3D printer (BioX, Cellink, USA). Scaffolds were fabricated at room temperature (23 °C). The nozzle size was 0.26–0.6 mm. The area of the samples was 2 cm × 2 cm. The number of layers was from 1 to 4. In the case of the material with a lower initial viscosity, it can be printed with a small nozzle (0.26 mm), low pressure (approximately 50 kPa), and average speed of printing unit (6 mm/s). In the case of samples with a higher initial viscosity, the 3D printing process requires a higher pressure (approximately 350 kPa), with average speed of printing unit (2 mm/s) with nozzle (0.6 mm). Hydrogels used for scaffold preparation were the same as described and characterized throughout the study.

### Solution/Hydrogel Characterization

#### Rheological Properties

To investigate the rheological properties of the various hydrogel systems, we used a rotational rheometer Anton Paar MCR 502 (Anton Paar, Austria) with parallel plate geometry, where the upper plate has a 25 mm diameter. The required temperatures were achieved by using a Peltier cell (Anton Paar, Austria).

For the basic rheological tests as flow curve or thinning, the shear mode was used to determine the rheological performance of the prepared systems as well as the effect of CI. For shear thinning (ST) tests, five cycles were set. In this examination, strain deformation alternates regularly from 0.1 to 100% counted as one cycle. The testing was carried out at room temperature. For the cross-linking process, investigation in oscillation mode was used to identify the moment when the storage modulus overcomes loss one and thus clearly calculate the time needed for considerable cross-linking. The frequency used for this investigation was set to 1 Hz and strain deformation to 1%, and viscoelastic moduli were collected over time. All measurements were performed at 25 °C and performed three times. The error bars for these measurements are smaller than the size of the symbols.

#### Magneto-Rheological Investigation

The magneto-rheological properties were investigated using a rotational rheometer Physica MCR502 (Anton Paar, Austria) equipped with a magneto-device (MRD 180/1T) and parallel-plate geometry (PP20/MRD/TI/S). In this case, the magnetic field strength (*H*, kA/m) is generated perpendicularly to the surface of the measuring geometry. The sample with a height of 1.25 mm was placed between the plates and subjected to oscillatory shear. For the ST tests, six cycles were set. The strain deformation alternates regularly from 0.1% to 100% and at a frequency of 1 Hz. The three cycles were performed in the absence of a magnetic field and the other three in the presence of a magnetic field with a magnetic field strength of 832 kA/m. All measurements were performed at 25 °C and done three times. The error bars for these measurements are smaller than size of the symbols.

#### Cytotoxicity

The test of cytotoxicity was provided by EN ISO 10993-5 with mouse fibroblast cell line NIH/3T3 (ECCAC 93061524). Dulbecco’s modified eagle’s medium (Biosera, France) supplemented by 10% of calf serum (Biosera, France) and 1% of penicillin/streptomycin solution (Biosera, France) was used as a culture medium. The culture conditions were set to 37 °C with a humidified atmosphere of 5% CO_2_ in the air. Cells were seeded into 96-well plates in the concentration 1 × 10^5^ cells/mL, which equals to the number of 1 × 10^4^ cells per well. Cell plates were incubated for 24 h.

At the same time, the hydrogel extracts were prepared according to EN ISO 10993-12. To the tested hydrogels, the culture medium was added so that the concentration corresponded to 0.1 g of particles/mL of medium. Then it was placed in a shaker for 24 h at 450 rpm and 37 °C. Parent extracts (100%) were then diluted in the medium to obtain concentrations of 90, 75, 50, 25, and 10%. On the second day, the medium in the 96-well plates was aspirated (after 24 h of cell incubation) and replaced with a 100 μL of the extract dilutions series; reference wells were filled with the pure culture medium without extract. The cells with extracts were incubated for another 24 h. All measurements were performed in quadruplicates.

After 24 h of exposure, the extracts were aspirated. Next, the medium containing 0.5 mg/mL of 3-[4,5-dimethylthiazol2-yl]-2,5-diphenyltetrazolium bromide (MTT—Molecular Probes, USA) were added to each well. The cells were stored for an additional 4 h in the incubator. During this time, MTT was metabolized to formazan. The medium with remaining MTT was aspirated and 100 μL of dimethyl sulfoxide (Molecular Probes, USA) was added to dissolve formazane, which was allowed to work for 15 min. The resulting coloration was measured by a microplate reader Infinite M200PRO (Tecan, Switzerland) at a wavelength of 570 nm, with the resulting absorbance corresponding to the number of living cells. Results are presented as cell viability after extract exposure relative to reference cells cultivated without extract. The reference was set to 1 and corresponds to 100% cell survival. Values >0.7 indicate the no-cytotoxicity effect, and values <0.7 indicate a cytotoxic effect.

#### Swelling Properties

The hydrogels in the form of a disk, 15 mm in diameter and 1 mm thickness, were immersed in deionized water, and the mass was determined along the time using analytical balance. Before measuring, the sample was picked out of the water and excess water was wiped with kimwipe paper. The equilibrium water content (EWC) in weight percent expresses the maximum amount of the water swollen to the hydrogel at given condition and can be calculated from eq 1.
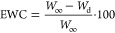
1where *W*_∞_ is the weight of the swollen hydrogel and *W*_d_ is the weight of the lyophilized one.

The rate of the hydrogel water sorption is expressed by the sorption degree (SD) and can be determined from eq 2.
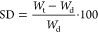
2where *W*_t_ is the weight of hydrogel at different time intervals and *W*_d_ is weight of the lyophilized sample.

In the case of study of the water transport mechanisms for the hydrogel-based sample, the Ritger–Peppas model (eq 3 was used for fitting the experimental data.
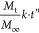
3(*M*_t_/*M*_∞_) is fractional water content and *M*_∞_ is weight of the measured sample at EWC and *M*_t_ is at a certain period of time, *k* is a kinetic constant, *t* is the diffusion time, and *n* is the time exponent that can be related to the solute transport.

The diffusion coefficients are calculated according to eq 4
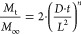
4*D* is the diffusion coefficient, *t* is the time of diffusion, *n* is the time exponent that can be related to the solute transport, and *L* is the thickness of the thin disk. These calculations are restricted to use only for thin hydrogel films/disks. Thus, in our case, the utilization of the samples with diameter to thickness ratio of 15 to 1 meets these requirements.

#### Computed Tomography

To explore the inner structure and porosity of the chosen cross-linked solutions in the bulk form, we used computed tomography (Skyscan 1174, Bruker). All tested samples used for investigation were in an EWC. The device was equipped with an X-ray power source (at the voltage of 20–50 kV and maximum power of 40 W) and an X-ray detector. The CCD 1.3 Mpix unit was coupled to a scintillator by a lens with a 1:6 zoom range. Projection images were taken at angular increments of 0.3° at a tube voltage of 35–40 kV and current of 585–730 μA. Duration of exposure was set to 15–30 s without the use of a filter. 3D reconstructions were created via the installed CT image analysis software (version 1.16.4.1, Bruker, USA). The results, in terms of total porosity, are expressed as the average and standard deviations determined from three different cylindrical sections. The representative cross-sectional images of 2.27 mm diameter and 3 mm height were exported from DataViewer software.

### Scaffold Characterization

#### Microscopy (Optical and Electron with EDX)

To identify the dispersity of CI in the bulk and observe a porosity level, scanning electron microscopy (SEM) (VEGA 2 LMU, TESCAN) was used. The samples were swelled to EWC, then kept in freezer for 24 h at −20 °C, and then 48 h lyophilized. Cross-section for SEM investigation was prepared using the brittle crack of the sample after lyophilization. The images were acquired in backscattered electron (BSE) and secondary electron modes. All samples were properly dehydrated and coated with a Au/Pd powder. In order to observe cell proliferation, a confocal laser scanning microscope [FV3000 (Olympus, Japan)] was used.

#### Magnetic Activity of Fabricated Scaffolds

For the examination of scaffold magnetic response, an electromagnet providing a magnetic field strength in the range (0–140 kA/m) and changing on/off cycles was used, and the schematic illustration of this setup is shown in Figure S2. The pictures of scaffolds and their motion shown in Videos S1 and S2 were captured using a Xiaomi Redmi 10 camera.

#### Cell Viability and Proliferation

The mouse fibroblast cell line NIH/3T3 (ECCAC 93061524) in a suspension at a concentration of 2 × 105 per 1 mL was seeded on the scaffolds with dimensions of 1 × 1 cm placed in 35 mm cell culture dishes. Culture medium and conditions were as described before. The cells were allowed to proliferate on the sample for 3 days. After this period, the cells on the samples were fixed and permeabilized. Cells were fixed using 4% formaldehyde (Penta, Czech Republic) for 15 min, washed by phosphate buffered saline (PBS - Biosera, France), and subsequently poured with 0,5% Triton X-100 (Sigma-Aldrich, USA) for 5 min to permeabilize. After this time, the cells were washed three times by PBS. Subsequently, the DNA within nuclei was stained using 5 μg/mL Hoechst 33258 (Invitrogen, USA) and the actin in a cytoskeleton was dyed using two drops of ActinRed 555 (Thermo Fisher Scientific, USA) per mL of PBS. After 30 min in the dark, cells were washed by PBS. Morphology of cells was recorded using an Olympus FV3000 confocal laser scanning microscope (Olympus, Japan).

## Results and Discussion

Hydrogel scaffolds based on HA and CI particles were prepared. Prior to 3D printing, the cross-linking procedure was optimized for bulk hydrogel samples. The cross-linking system was designed with respect to an optimal safe time period (processing window), i.e., the time necessary for mixing all reagents and printing the scaffold prior the formation of cross-linked structure.

### Cross-Linking Reaction

**Figure 1:**
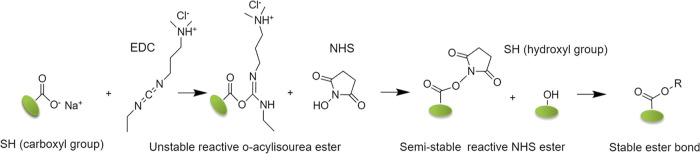
Possible cross-linking mechanism of the SH in the aqueous medium with slightly base pH ∼ 7.8.

The HA provides carboxyl and hydroxyl groups that occur naturally in the structure. They can be used for cross-linking. Numerous methods of chemical cross-linking of HA have been reported.^50,51^ In this work, EDC and NHS were used. This curing system has a low toxicity and is water-soluble.^39,52^ It allows for the formation of ester groups, as depicted in Figure 1.

The carboxyl groups are activated by EDC molecules to form an unstable *O*-acylisourea group. It is transformed to more stable reactive ester by nucleophilic reagent NHS. This structure supports the condensation reaction, giving rise to ester bond formation.^40,41^ The cross-linking conditions were optimized with respect to a safe time period (processing window), which is defined as time when the material does not provide any significant chemical, structural, and most importantly rheological difference and can be safely processed using 3D printing. Finally, the EDC/NHS ratio 1:1 w/w and pH 7.8 were selected.

### Rheological Properties

Before the hydrogels were prepared, the selection of a suitable polymer matrix for printing was performed. For scaffold fabrication, the polymer solutions have to provide specific rheological features, i.e., yield stress (YS) and ST. The initial screening of the rheological capabilities is presented in Figure S3, which shows various concentrations of sample HA2; Figure S4 shows those of sample HA3. It is clearly seen that 7 wt % HA concentration exhibiting certain YS for both of them is most reliable in connection to further 3D printing. The YS is further determined as a stress necessary to induce flow. It can be observed from the viscosity dependence of the shear stress or shear stress dependence on the shear rate (Figure S5). The HA aqueous solutions of three different molar masses were compared. While HA1 showed Newtonian liquid-like behavior with no YS, both HA2 and HA3 provided YS, which was increased with molar mass of HA. The YS of HA2 and HA3 is visualized in Figure S5a as YS_2_ and YS_3_, respectively. In Figure S5b, the YS can be observed as nonlinear (pseudoplastic) dependence of stress on the shear rate. This feature is the most pronounced in HA3.

**Figure 2:**
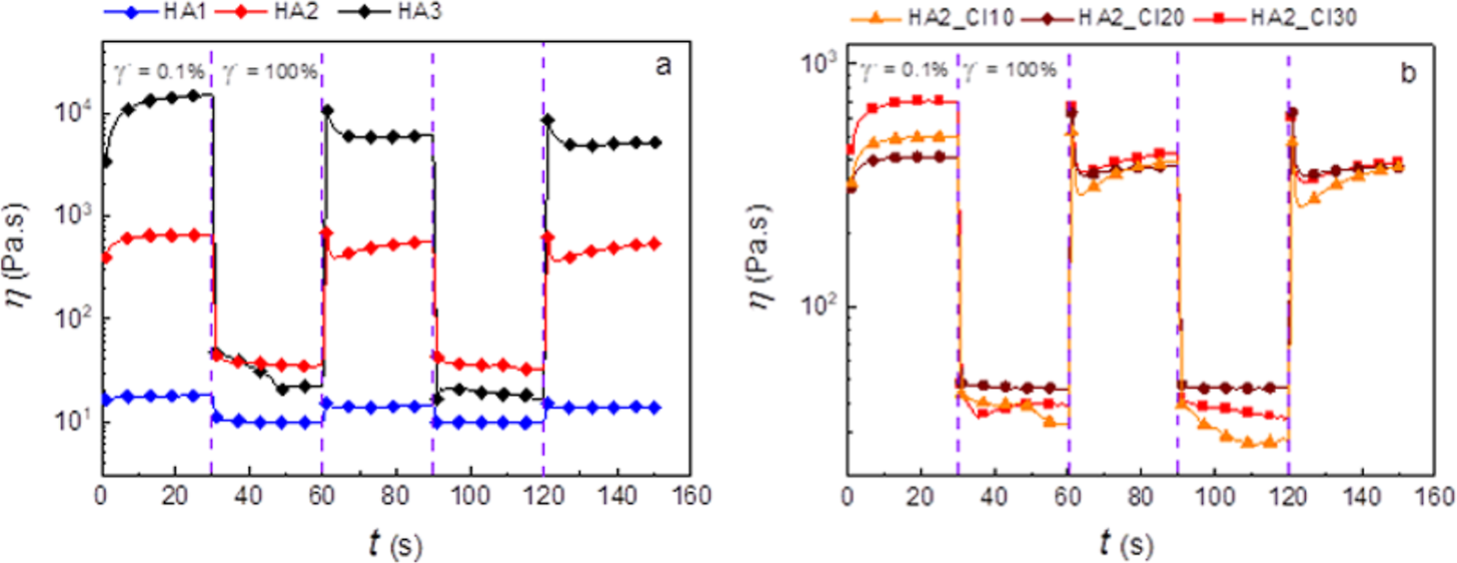
Rheological properties of HA aqueous solution and evaluation of ST. The time dependence of viscosity at the alternating frequency 0.1 and 100 s^–1^. (a) Three different molecular weights of HA (Table 1) were compared and are labeled as HA1, HA2, and HA3, respectively. (b) HA2 filled with carbon iron particles at concentrations 10, 20, and 30 wt %; samples are labeled as HA2_CI10, HA2_CI20, and HA2_CI30, respectively. In all experiments, the solutions of 7 wt % HA were used.

ST was recognized as another important rheological parameter of polymer solutions suitable for 3D printing. It is defined as a decrease in material viscosity upon application of strain. It reflects the ability of the polymer solution to pass the nozzle of the printer at high shear rates and keep the proper shape of printed scaffold at low shear rates. The higher the difference in viscosities at different shear rates developed, the better the processing of the materials that can be expected. In Figure 2a, the comparison of ST of three different HA aqueous solutions is depicted. While the HA1 shows only negligible viscosity changes in response to alternation shear strain, the HA2 and HA3 provide significant differences, which point them as suitable candidates for scaffold fabrication. In order to see how CI particles affect the initial flow behavior of CI–HA solutions, the optical image of various samples HA1 and HA2 with and without CI particles was shown (Figure S6). Furthermore, in Figure 2b, the effect of the addition of CI particles on ST is shown. The three different concentrations of CI (10, 20, and 30 wt %) were tested. All the samples exhibit similar dependence; even at the highest CI concentration (HA2_CI30), the ST ability is retained.

The results obtained from investigation of YS and ST of HA solutions (Figures S5 and 2) pointed to HA2 and HA3 as promising polymers for scaffold fabrication, and thus these two HA types were used in further experiments. The cross-linking kinetic was optimized using rheological measurements. The storage (*G*′) and loss (*G*″) modulus were measured in time, as shown in Figure S7. The cross-linking is associated with the changing rheological response from liquid-like to solid-like behavior. In Figure S7a, the cross-linking kinetics of HA2 is shown. The storage modulus starts to dominate over loss modulus after ∼750 s, as is seen in the inset graph. The presence of 30 wt % of CI particles shifted the crossover point to ∼5700 s, i.e., ∼ 95 min, which is a comfortable processing window (Figure S7b). The utilization of the same amount and ratio of components of the cross-linking system did not result in network formation of HA3 in the presence of 30 wt % CI (Figure S7c); even the elevated temperatures were used to initiate the cross-linking reaction. However, prolongation of the reaction time and increase of reaction temperature was not sufficient to obtain the cross-linked network.

### Magnetorheology

**Figure 3:**
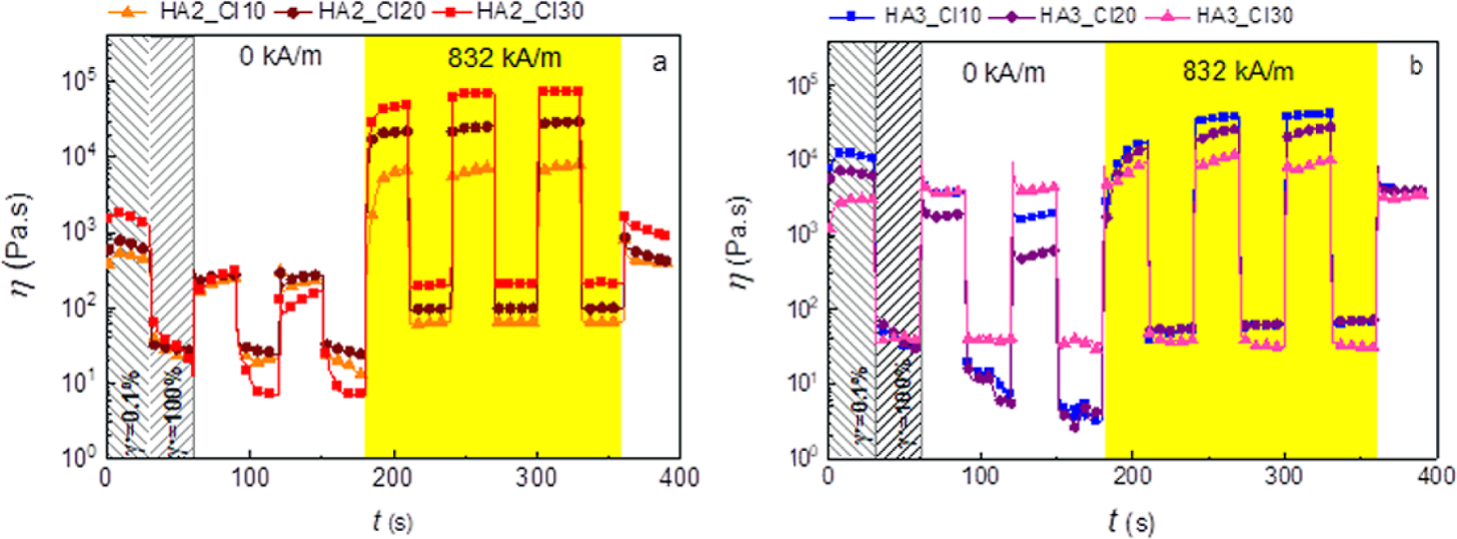
ST in the presence of the alternating magnetic field for the 7 wt % HA solutions. (a) HA with the 377 kDa molar mass filled with 10, 20, and 30 wt % of CI; samples are labeled as HA2_CI10, HA2_CI20, and HA2_CI30, respectively. (b) HA with the 978.6 kDa molar mass filled with 10, 20, and 30 wt % of CI; samples are labeled as HA3_CI10, HA3_CI20, and HA3_CI30, respectively.

Figure 3 provides information about behavior upon the cyclic switching on and off the magnetic field for the samples based on the molecular weight 377 kDa (HA2) and 978.6 kDa (HA3) with different CI amounts. Both systems have fast and reversible response to the magnetic stimulus over the on/off cycles. Moreover, the higher CI content is able to increase the sample response to the magnetic field. In the case of HA2, the difference between the viscosities of the sample containing 10 wt % CI (HA2_CI10) and 30 wt % CI (HA2_CI30) is more than 1 order of magnitude higher in the presence of the magnetic field and reaches 10 and 100 kPa s, respectively. The system based on HA3 does not provide such huge magneto-responsive characteristics due to the higher off-state viscosity. This behavior is analogous to the typical MR suspensions^54^ and elastomers.^55^ Moreover, a similar behavior was also observed for the superparamagnetic hydrogels, however, with incomparably smaller MR activity changing its viscosity in the same order of magnitude when the magnetic field was applied with values ∼100 Pa.^56^ These results clearly indicate that the presence of the magnetic particles provides additional functionality to this material upon an external magnetic field stimulus.

### Cytotoxicity of Bulk Hydrogels

**Figure 4:**
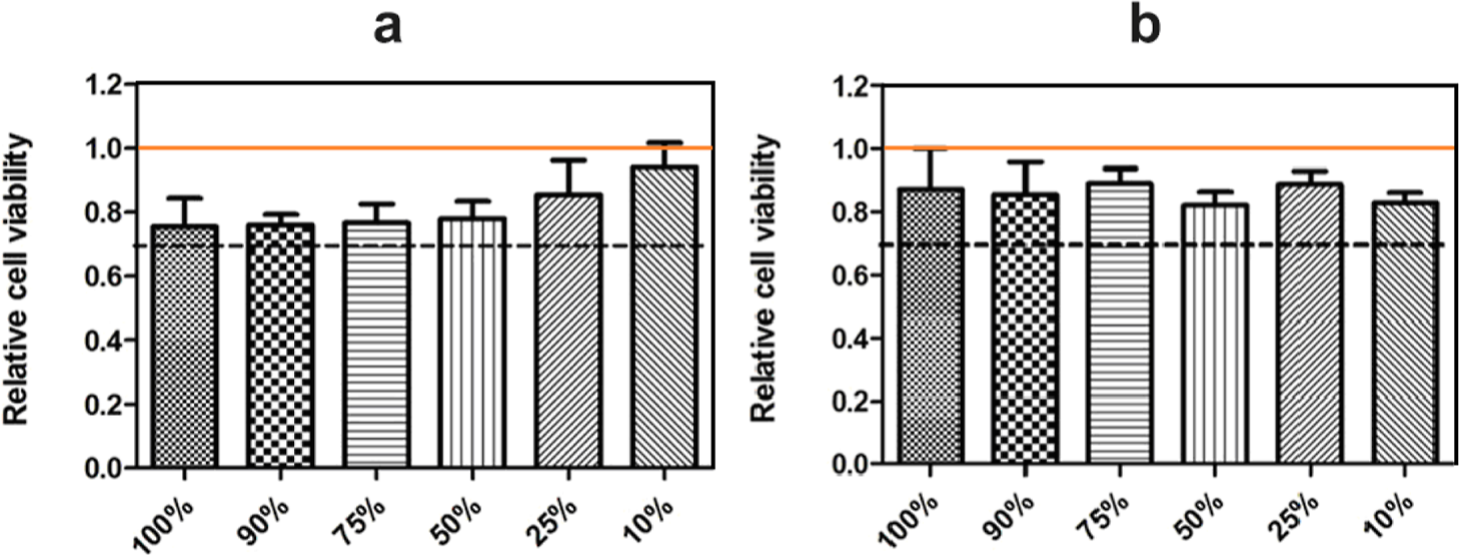
Relative cell viability in the solution concentration from 10 to 100% of the original hydrogel. (a) HA2 (7 wt % solution) of HA and (b) HA2_CI30 (7 wt % solution and 30 wt % of CI particles). The orange solid line represents the reference of the cell culturing solely in the cultivation medium. The black dashed line represents the line between cytotoxicity (below) and noncytotoxicity (above) of the investigated hydrogel systems.

In order to evaluate the possibility of the presented magnetic hydrogels as scaffolds for cell cultivation, the hydrogels in bulk were investigated by the standard cytotoxicity test. As can be seen, in all cases (Figure 4), relative cell viability is not lower than 0.7 and lower values were not observed for all solution concentrations. This clearly indicated that the mentioned hydrogels are totally noncytotoxic and safe for further cell contact. The most promising system (HA2_CI30) has one of the highest levels of cell viability. Also, it is notable that the system without CI content has lower cell viability properties. It is possible that CI has a role not only as a magnetic filler but also as cell support. Therefore, the sample with HA2_CI30 was used for 3D printing of magneto-responsive scaffolds as is elaborated below in the text.

### Swelling of Fabricated Magnetic Hydrogels

The swelling properties of hydrogels belong to the basic characteristics of hydrogels. It shows the ability of the materials to absorb water and thus also change the shape accordingly. In the case of scaffolds, any dimensional changes are not desirable. It is expected that the printed structure will serve in the as-printed shape. The addition of particles to hydrogel affects the cross-linking kinetics, the density of the formed network, and thus also swelling. The swelling properties were investigated with respect to EWC, SD, and water diffusion mechanism.

#### Equilibrium Water Content

EWC is determined as the maximal amount of water absorbed with the hydrogel at the given conditions. The effect of CI content on EWC of HA2 hydrogels is shown in Table 2. The neat HA2 hydrogels provided the highest EWC value, 98.6%. The addition of 10 wt % of CI (HA2_CI10) resulted in the EWC value decreasing to 94.2%. The addition of 20 and 30 wt % of CI resulted in an increase of EWC values to 95.0% and 97.0%, respectively. It suggests that the density of cross-linking and structure of pores affected the particle content. The presence of particles hinders the network formation and facilitates the polymer network to absorb water. The effects of CI on porosity of hydrogels were further investigated. Here, it has to be noted that due to the fact that CI particles do not have any surface modification, they do not contribute to the cross-linking reactions, and swelling capability lies on the structural morphology of the hydrogels, which needs further tomography investigations.

**Table 2:** Swelling of Crosslinked HA2 Hydrogels with Various Contents of CI^a^

sample name	CI content (wt %)	EWC (wt %)	SD (%)	*D* (m^2^·s^–1^)	*n* (−)
HA2	0	98.6	6400	8.2 × 10^–10^	0.13
HA2_CI10	10	94.2	1600	8.2 × 10^–10^	0.13
HA2_CI20	20	95.0	1900	6.1 × 10^–10^	0.14
HA2_CI30	30	97.0	3200	4.2 × 10^–10^	0.19

a Summarized values of the EWC, degree of swelling in equilibrium (SD), diffusion coefficient (*D*), and time exponent of diffusion (*n*).

#### Sorption Degree

The rate of hydrogel water sorption is expressed by the SD. The effect of CI content on SD of hydrogels is depicted in Figure S8. The neat HA2 shows the fastest sorption; it provided 4000% SD within 90 min, and the equilibrium state was obtained at 6400% after 4000 min. The addition of CI particles resulted in lower SD compared with neat HA2. The values of SD after first 90 min do not differ significantly. The differences can be observed after 120 min, when the SD of HA2_CI30 increased more than twice compared to HA2_CI10 and HA2_CI20. This trend is observed also in the equilibrium state, where SD of HA2_CI30 reached 3200%, while those of HA2_CI10 and HA2_CI20 reached 1600% and 1900%, respectively.

#### Water Diffusion

To complete the swelling properties, the water diffusion kinetic was evaluated in terms of diffusion coefficient (*D*) and solute transport parameter (*n*). Both parameters can be obtained by fitting the time dependence of the fractional water content (*M*_t_/*M*_∞_) with the Ritger–Peppas model (eq 3), as shown in Figure S9. This model is applicable in the early stage of diffusion, up to 60% of EWC, and it is frequently used for the water transport mechanism in hydrogels.^57,58^

For a thin hydrogel film, there are two cases of water transport mechanisms, Fickian and Non-Fickian. Contrary to Fickian diffusion, the sharp boundary between the swollen and dry regions is present in non-Fickian diffusion systems. The water transport mechanism can be distinguished by parameter *n*. The Fickian diffusion can be observed in case of *n* = 0.5, while the anomalous non-Fickian diffusion can be found when 0.5 < *n* < 1. If the *n* ≤ 0.5, then the material diffusion obeys Fickian laws; however, the water diffusion rate is much less below the polymer chain relaxation rate. In this case, the transport mechanism is called as Fickian diffusion from the nonswellable matrix.^59^ The fitting of the experimental data with the Ritger–Peppas model showed that all the hydrogels obey the Fickian laws from the nonswellable matrix. The parameters are listed in Table 2. The values of the diffusion coefficient show that with the increasing content of CI particles, the diffusion is slower.

As can be seen in Figure S9, the neat HA2 hydrogel (Figure S9a) and HA2 filled with 10 wt % of CI (HA2_CI10, Figure S9b) showed very similar diffusion coefficients. It indicates that the water absorption is not affected significantly by CI in this first stage of absorption. With the further increase of the particle content to 20 and 30 wt % (HA2_CI20 and HA2_CI30, Figure S9c,d), the diffusion coefficient decreases significantly. As was mentioned above, this effect can be caused by different porosities of the material. Therefore, the computed tomography of bulk hydrogels was performed.

### Computed Tomography

**Figure 5:**
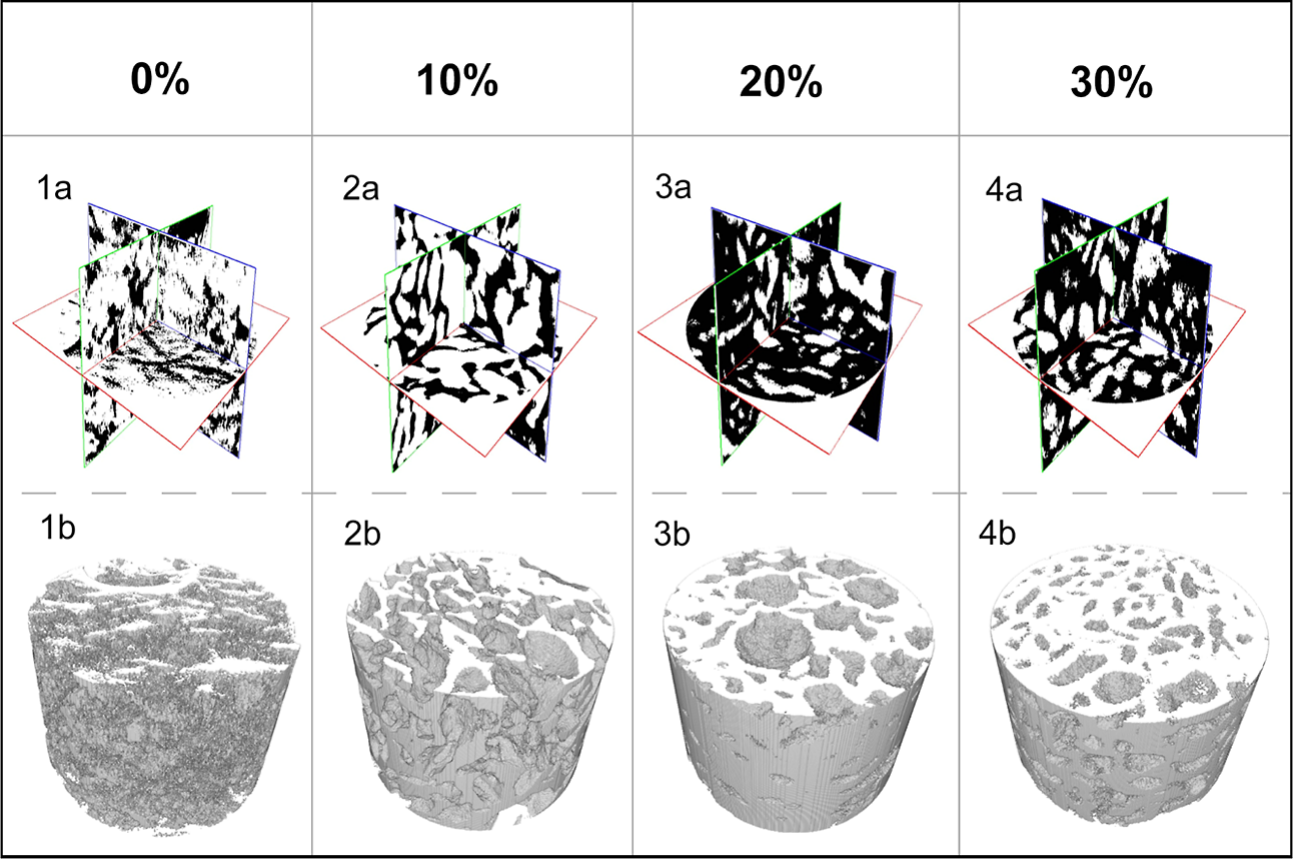
Tomography of the sample HA2 with different CI amounts (0, 10, 20, and 30 wt %). The row “a” (1a, 2a, 3a, 4a) demonstrates the structure cuts in three dimensions. The row “b” (1b, 2b, 3b, 4b) demonstrates the 3D model of the inner structure of the samples.

The tomography of bulk cross-linked hydrogels was performed to investigate the inner structure and porosity of the hydrogels. Magneto-active composite systems are usually observed using tomography,^60^ and also magneto-active hydrogels were investigated as well.^61^ The pore size and amount were affected by the CI content, as can be seen in Figure 5. The HA2 structure is formed by a high amount or small pores. They are rather open without significant boundaries (a–a and b–b). With the increasing CI content, the number of pores decreases and the size increases. The pores are regular, round-shaped, and closed (a–a, b and a–a, b). The difference is most obvious at HA2_CI30. This sample also exhibited the lowest diffusion, which can be associated with these structural changes.

### Microscopy

The internal structure and porosity of the hydrogel were correlated with SEM. In Figure S10a–d, the brittle cracked areas of lyophilized samples are shown. The pores of neat HA2 are open, and the structure is irregular. The presence of CI made the pores smaller and more closed. Although the SEM images show surface deformation during formation of a brittle crack, the results very well correlate with CT observations. In backscattered mode (Figure S10e–h), the particles distribution in the hydrogel matrix is visualized. The CI particles are observed as white spots. The distribution of the particles within the matrix is homogeneous.

The detailed SEM image in Figure S11 shows the adhesion of the matrix to particles. The particles are wetted with the matrix well. The particles are present in the form of small clusters and in fact in the form they are supplied from the producer.

### Scaffold Types

**Figure 6:**
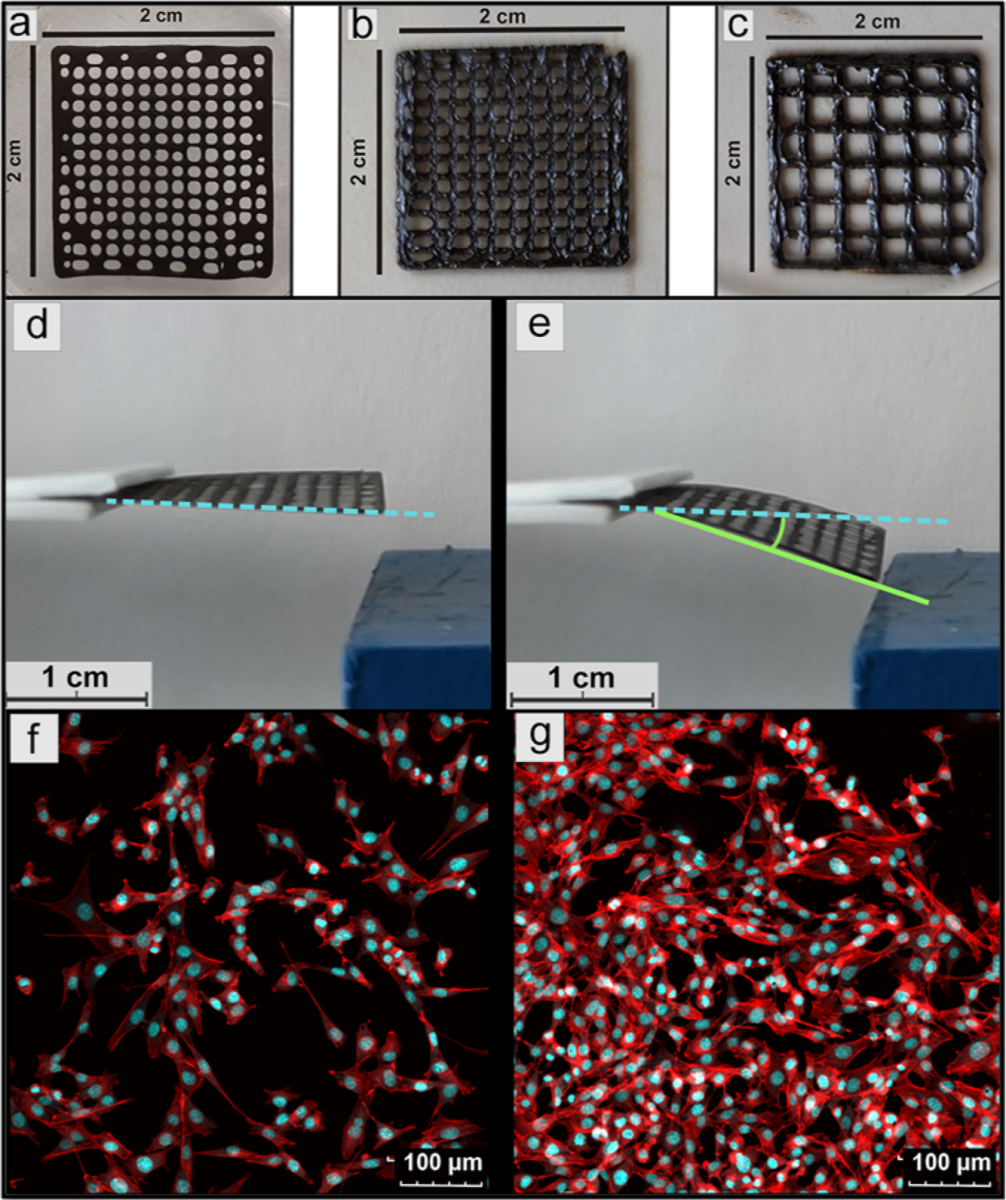
Scaffolds with different amount of layers fabricated using various types of nozzle width, (a) 1-layer 0.26 mm nozzle width, (b) 4-layer 0.41 mm nozzle width, and (c) 2-layer 0.6 mm nozzle width. Activity of the scaffold HA2_CI30 (d) in the absence and (e) in the presence of an external magnetic field strength of 140kA/m. Cell proliferation; (f) reference and (g) cells in the presence of a scaffold.

As can be seen in Figure 6, the selected concentration of HA as well as CI particle is suitable for 3D printing. The form and texture can be modified by software setting of a 3D printer. The 3D printing capabilities as well as shape fidelity were investigated using Leica software for images (Figure S12). For the scaffold printed using a 0.26 mm nozzle (Figure 6a) and set gap size 0.5 mm, the obtained individual filament thickness was 0.22 ± 0.01 mm with the real gap size between filaments of 0.53 + 0.01 mm (Figure S12a). In the case of scaffold printed using a 0.41 mm nozzle (Figure 6b) and set gap size 0.55 mm, the individual filament thickness was 0.40 ± 0.01 mm with the real gap size between filaments of 0.57 + 0.01 mm (Figure S12b). The last experiment was for scaffold-printed with a 0.6 mm nozzle (Figure 6c) and set gap size 1 mm; the individual filament thickness was 0.63 ± 0.006 mm with the real gap size between filaments of 1.37 ± 0.005 mm (Figure S12c). Here, it can be concluded that 3D printed hydrogel scaffolds provide excellent printed capabilities and very good shape fidelity, especially for a sample printed with nozzle 0.26 and 0.41 mm width, due to the good printing capabilities of the prepared formulations. The hydrogel scaffolds fabricated using a nozzle with a 0.6 mm width have limitations in shape fidelity; however, they still provide very promising results significantly better than those already published for similar systems.^62,63^ Moreover, the dispersion of CI particles was investigated on the printed scaffolds, and as can be seen in Figure S13, they have very good (homogeneous) dispersion in the scaffold after the 3D printing procedure.

As can be seen in Figure S14, a scaffold tends to increase its volume in the water environment significantly. However, after 3 days in ultrapure water and at the room temperature, the original size increased more than twice. The important note here is that the scaffolds still copy the initial printed structure precisely and no mechanical deformations are visible due to such significant swelling.

### Magnetic Activity

After several experiments, it was found that the minimal CI amount for suitable magnetic sensitivity should be at least 30 wt %. The scaffold with 30 wt % of CI shows reliable mechanical stability and required response to the magnetic field application (Videos S1 and S2). The actual magneto-actuation angle of the various scaffolds is presented in Table 3. Scaffold with the lowest CI content (10 wt %) HA2_CI10 does not react on the magnetic field. The second scaffold (HA2_CI20) with 20 wt % of CI was capable of reacting on the 70 and 140 kA/m magnetic field, showing 5 and 7°, respectively. The highest actuation performance induced by magnetic field exhibited scaffold HA2_CI30, which showed 9 and 12° for magnetic field strengths 70 and 140 kA/m, respectively. This scaffold is also present in the absence of a magnetic field (Figure 6d) and in the presence of 140 kA/m (Figure 6e).

**Table 3:** Summarizing Table of the Angle Changes upon Various Magnetic Field Strengths

	magnetic field strength (kA/m)			
		10	70	140
CI content (%)	10	0°	0°	3°
	20	0°	5°	7°
	30	0°	9°	12°

### Cell Proliferation

This test was related to the most reliable and suitable scaffold type (377 kDa, 7 wt % SH, 30 wt % CI) for the final goal. As can be seen in Figure 6g, the presence of scaffold significantly supports cell (the mouse fibroblast cell line NIH/3T3) growth, even more than what is shown in Figure 6f. The reason could be the original HA nontoxic nature,^35,64^ as well as nontoxic CI.^26,27^ In addition, in only 4 days, cells were able to adhere in the scaffold structure and proliferate. All mentioned information makes the chosen scaffold the best candidate for further in vitro experiments.

## Conclusions

In this paper, solutions based on the combination of natural polymer HA acid and CI were successfully produced. The most promising hydrogel formulation was the one with HA of the molecular weight 377 kDa, with a concentration of 7 and 30 wt % amount of CI labeled HA2_CI30. This system showed pseudoplastic behavior with remarkable YS above 10^3^ Pa. Such properties are beneficial for 3D printing. The CI particle incorporation into the HA matrix and the porosity was examined using SEM and CT. This provided information about descent particles incorporation and their considerable influence on the pore amount and size. According to the results, the higher the amount of CI present, the higher the hydrogel porosity and the better the pore distribution in the hydrogel system that can be obtained. These findings are in good agreement with the results from swelling testing, where the highest swelling degree (3200%) among the filled samples achieved the sample with 30 wt % CI amount. Also, the bulk hydrogels were tested for cytotoxicity and their nontoxicity to the NIH/3T3 type of cells was proved. Such a reliable type of hydrogel was further investigated from the 3D printing point of view. The scaffold is well printable due to high YS, provides good shape fidelity, and is suitable for further manipulation. 3D printed scaffold with 30 wt % of CI is magnetically sensitive and able to reversibly respond to low magnetic field strength 140 kA/m. Finally, the magnetically active scaffold can be applied as a suitable platform for cell in-growth and proliferation of NIH/3T3 cells. The investigated facts presented in this study support the idea of the significant potential of this type of scaffold system with very promising capabilities in the field of tissue engineering.

## Supporting Information Available

Schematic illustration of the cross-linked hydrogel preparation procedure; schematic illustration of the measuring setup for investigation of magnetic activity of fabricated scaffold by using alternating switching on/off cycles using an electromagnet; dependence of shear stress on the shear rate for different HA concentrations with molecular weight 978.6 kDa; dependence of the viscosity on the shear stress for different HA concentrations with molecular weight 377 kDa; rheological properties of HA aqueous solutions, evaluation of YS, shear stress dependence of viscosity, and shear stress dependence of shear rate; comparison of three different molecular weights of HA, image of different hydrogel formulations; time dependence of storage (*Ǵ*) and loss modulus (*Ǵ́*), the cross-linking kinetic investigated by rheology of HA aqueous solutions; dependence of the degree of sorption on the time for cross-linked hydrogels containing various amounts of CI; dependence of the water fractional content (*M*_t_/*M*_∞_) on the time for cross-linked hydrogels containing various amount of CI: (a) neat HA2, (b) HA2_CI10, (c) HA2_CI20, and (d) HA2_CI30; SEM images; distribution of CI particles in the HA2 matrix; investigation of the shape fidelity of the printed scaffolds fabricated using various types of nozzle width; and scaffold prepared from formulation HA2_CI30 (a) and part of this scaffold after 4 days of swelling (b) (PDF)
Magneto-responsive activity of 3D printed scaffolds (MP4)
Vertical movement of the scaffolds under applied magnetic field (MP4)
