# Supplementary material for: Smart Biopolymer Scaffolds Based on Hyaluronic Acid and Carbonyl Iron Microparticles: 3D Printing, Magneto-Responsive, and Cytotoxicity Study
Source: ACS Appl Bio Mater. 2024 Oct 17;7(11):7483–93. doi: 10.1021/acsabm.4c00567 (PMC11577426; doi:10.1021/acsabm.4c00567)
Supplement: Supplementary file 1 — mt4c00567_si_001.pdf [file mt4c00567_si_001.pdf]

Supporting information

Smart Biopolymer Scaffolds based on Hyaluronic Acid and Carbonyl Iron Microparticles: 3D printing, Magneto-Responsive and Cytotoxicity study

Danila Gorgol[1], Miroslav Mrlík[1,*], Filip Mikulka[2], , Zdenka Víchová[1], Leona Mahelová[1], Markéta Ilčíková[1,2,3] Antonín Minařík[1,2]

[1] Centre of Polymer Systems, Tomas Bata University in Zlin,Trida T. Bati 5678, 760 01 Zlin, Czech Republic

[2] Department of Physics and Materials Engineering, Faculty of Technology, Tomas Bata University in Zlin, Vavřečkova 275, 70 01 Zlin, Czech Republic

[3] Polymer Institute, Slovak Academy of Sciences, Dubravska cesta 9, 845 45, Bratislava 45, Slovakia

* corresponding author

e-mail of corresponding author: mrlik@utb.cz;

Electronic Supporting information

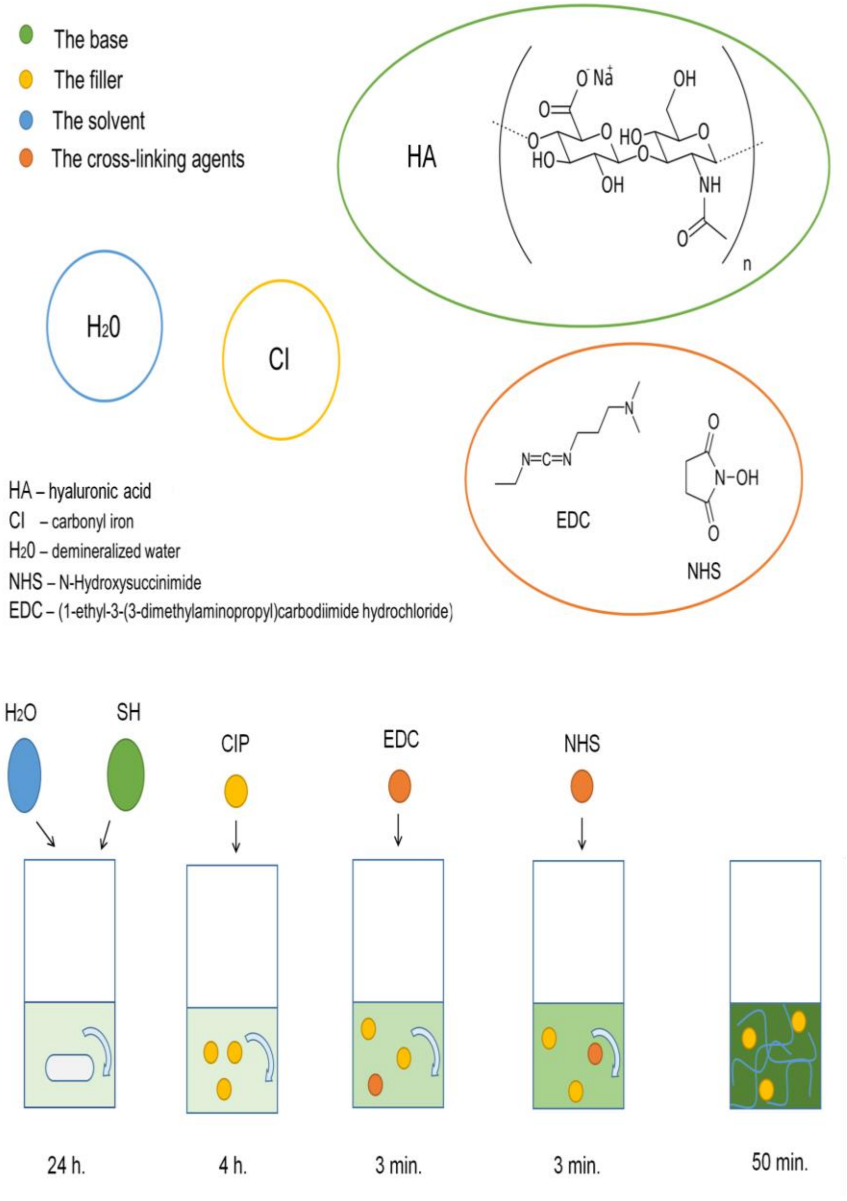

Figure S1. The schematic illustration of crosslinked hydrogels preparation procedure.

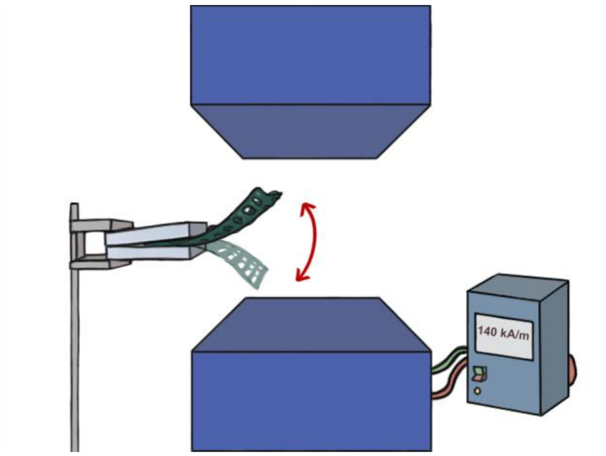

Figure S2: The schematic illustration of the measuring setup for investigation of magnetic activity of fabricated scaffold by using alternating switching on/off cycles using electromagnet.

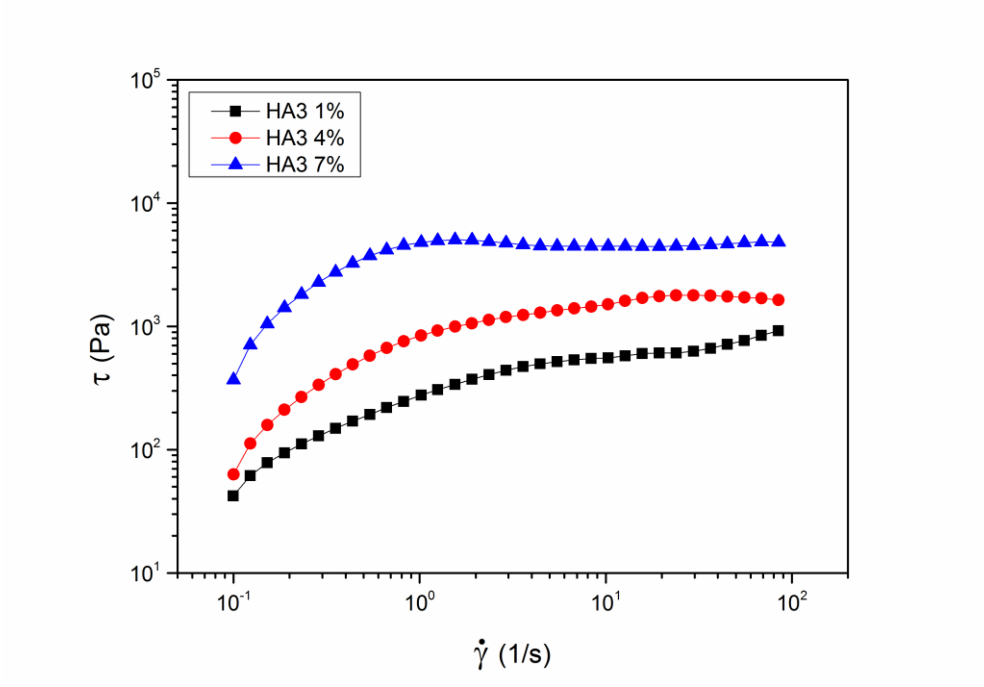

Figure S3: The dependence of shear stress on the shear rate for different HA concentrations with molecular weight 978.6 kDa.

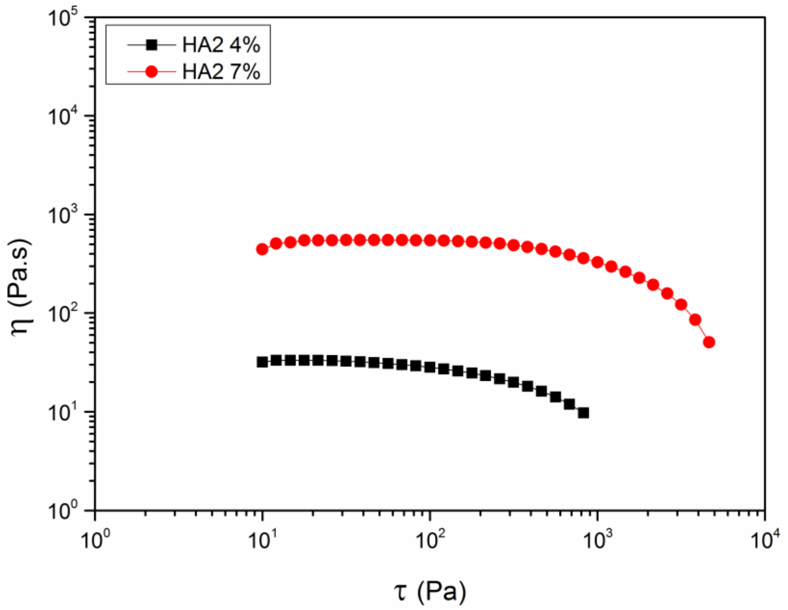

Figure S4: The dependence of the viscosity on the shear stress for different HA concentrations with molecular weight 377 kDa.

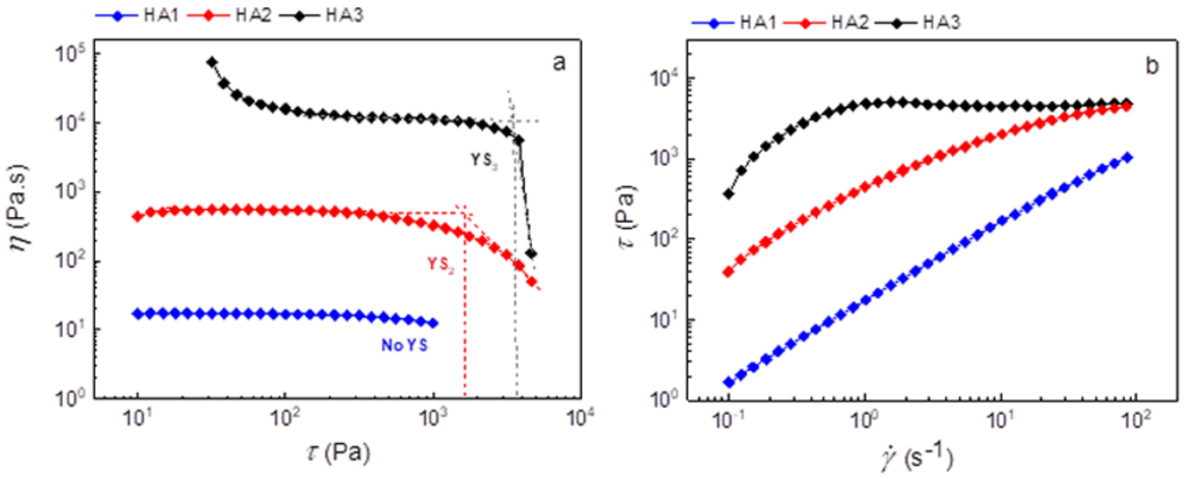

Figure S5: The rheological properties of HA aqueous solutions, evaluation of yield stress. a) dependence of the viscosity on the shear stress b) Dependence of the shear stress on the shear rate.

Three different molecular weight HA (Table 1) were compared, and are labeled as HA1, HA2 and HA3, respectively. In all experiments the solutions of 7 wt.% HA were used.

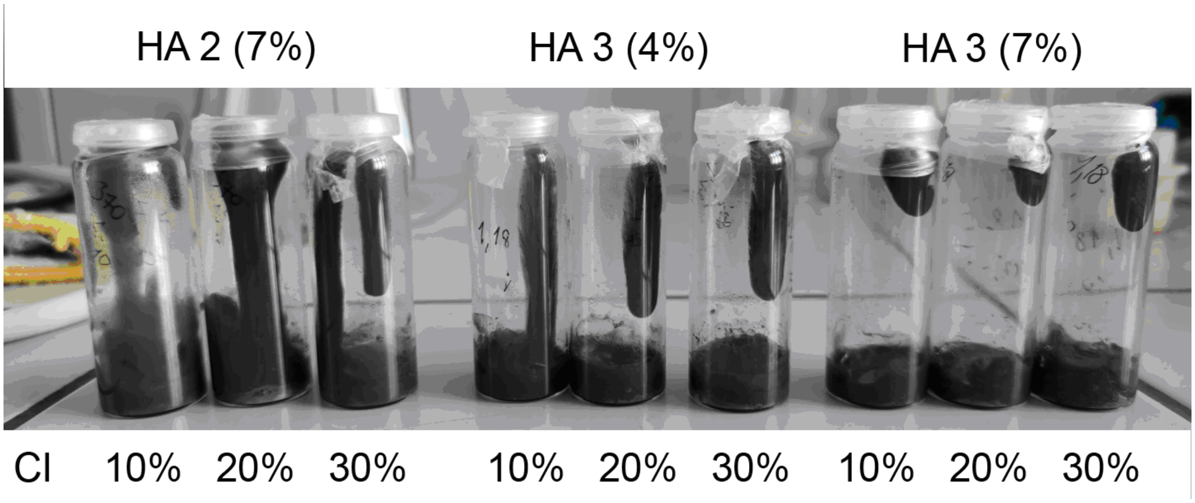

Figure S6: Different solutions without cross-linking

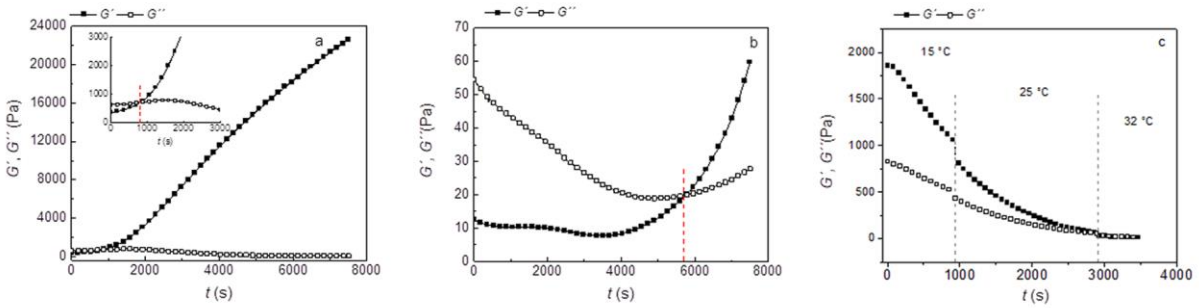

Figure S7: The time dependence of storage ($G´$) and loss modulus ($G´´$), the cross-linking kinetic investigated by rheology of HA aqueous solutions. a) HA2 b) HA2_CI30. c) HA of molar mass HA3_CI30. The 7 wt.% aqueous solution of HA, and same amount and ratio of cross-linking agents was used in all experiments.

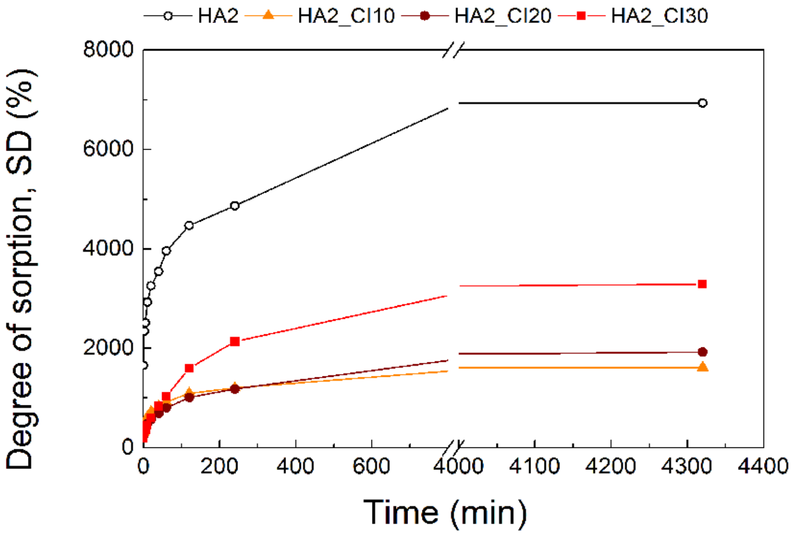

Figure S8: Dependence of the degree of sorption on the time for cross-linked hydrogels containing various amounts of CI.

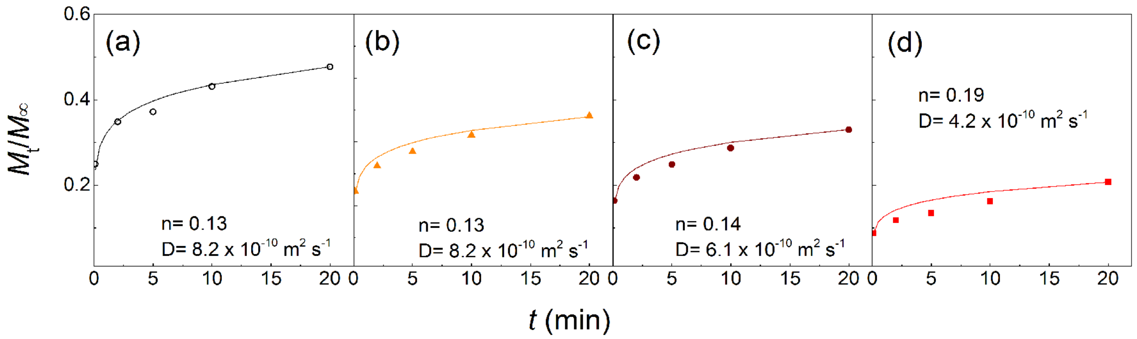

Figure S9: Dependence of the water fractional content (Mt/M∞) on the time for cross-linked hydrogels containing various amount of CI (a) neat HA2, (b) HA2_CI10, (c) HA2_CI20 (d) HA2_CI30.

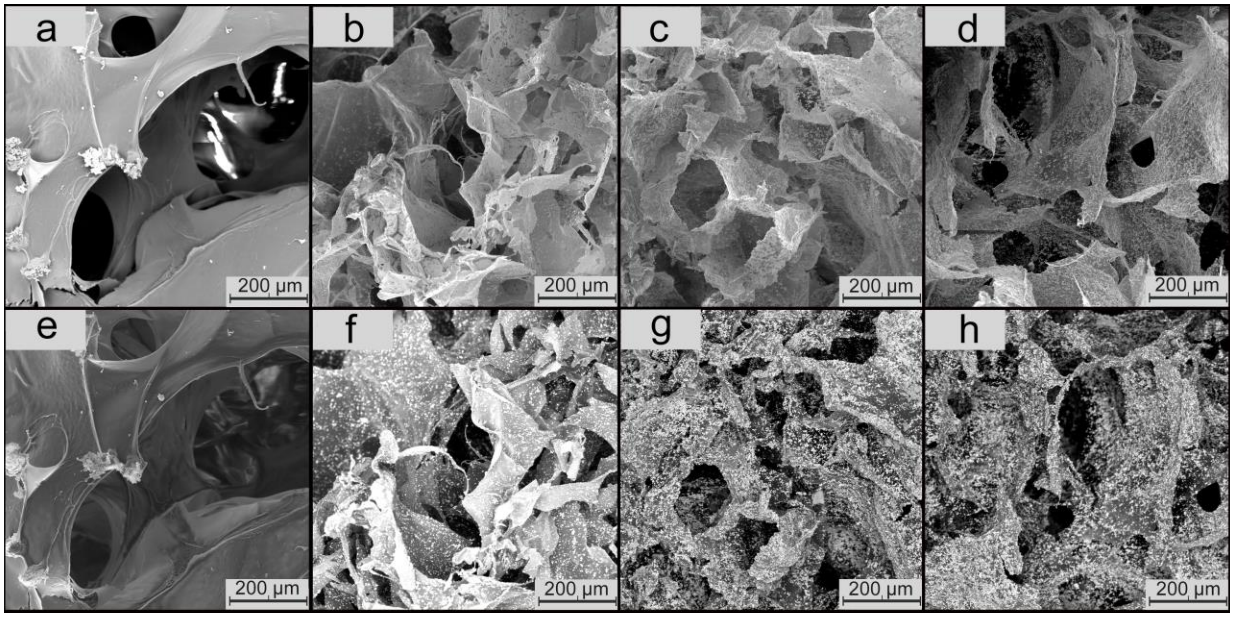

Figure S10: SEM images of HA2 crosslinked hydrogel with various CI content. a) neat HA2, b) HA2_CI10, c) HA2_CI20, d) HA2_CI30. SEM images in BSE mode e) neat HA2, f) HA2_CI10, g) HA2_CI20, h) HA2_CI30.

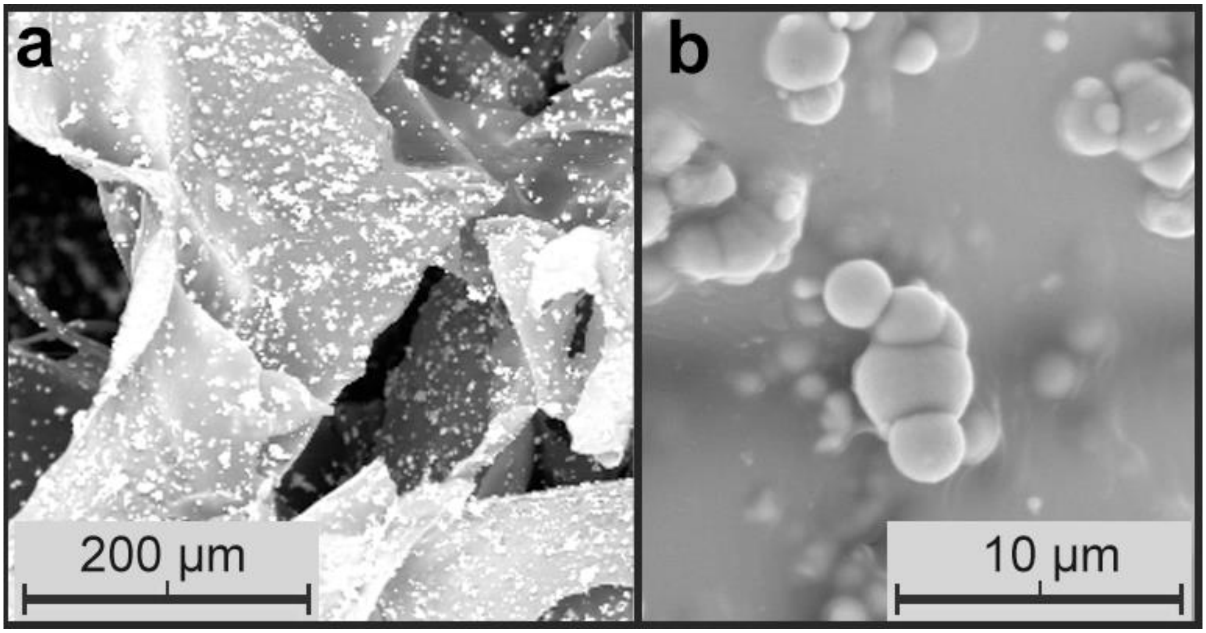

Figure S11: SEM image of HA2_CI30, cross-section of sample. a) distribution of CI particles in HA2 matrix visualize in SEM BSE mode, b) SEM image of CI particles in HA2 matrix, the good adhesion of polymer to particles.

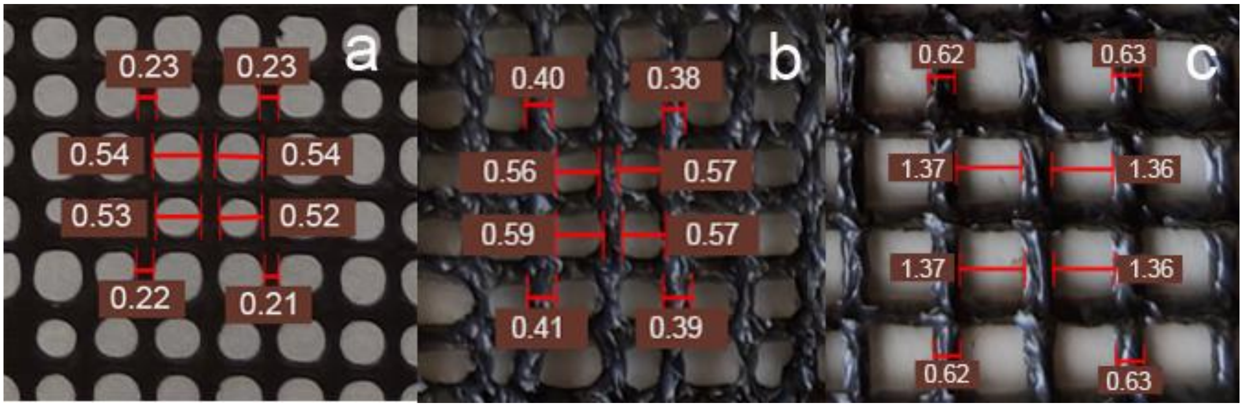

Figure S12: Investigation of the shape fidelity of the printed scaffolds fabricated using various types of nozzle width, a) 1-layer 0.26 mm nozzle width, b) 4-layers 0.41 mm nozzle width, c) 2-layers 0.6 mm nozzle width. All numbers in figure are in mm.

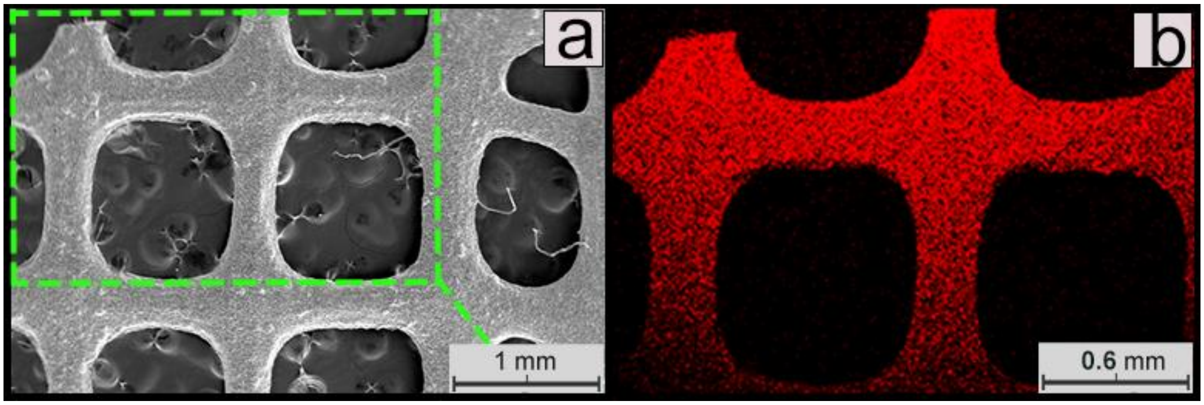

Figure S13: (a) SEM image of scaffold consisting of HA 2 with 30 wt.% CI, (b) Iron mapping using SEM-EDX, where green part is zoomed and red dots representing the presence of Fe particles.

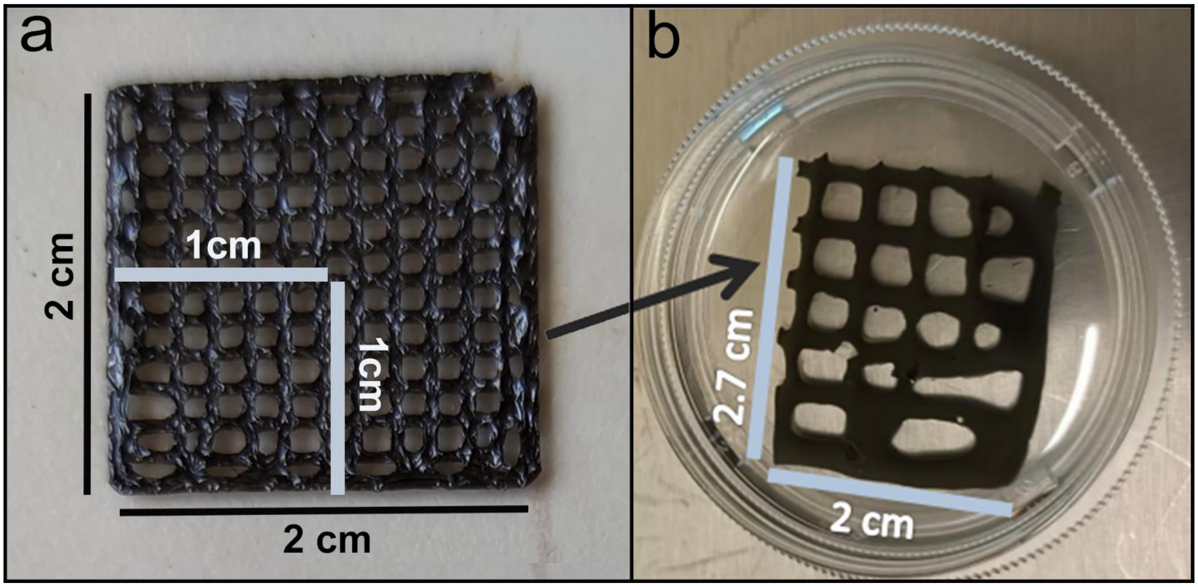

Figure S14: Scaffold prepared from formulation HA2_CI30 (a) and part of this scaffold after 4 days of swelling (b).

Video S1:Magneto-responsive activity of 3D printed scaffolds

Video S2: Vertical movement of the scaffolds under applied magnetic field.
